# Bioinspired functional materials for biomedical applications

**DOI:** 10.3389/fbioe.2026.1794001

**Published:** 2026-08-07

**Authors:** Yi Wu, Sihang Ren, Dan Zhang

**Affiliations:** Department of Plastic and Aesthetic Surgery, The Second Hospital of Dalian Medical University, Dalian, China

**Keywords:** bioceramic, bio-inspired materials, biomimetic implants, disease treatment, hydrogels

## Abstract

Bio-inspired materials, derived from the structures and functions of natural organisms, have shown significant promise in biomedical applications. This review highlights recent advances in three major classes of bio-inspired functional materials-hydrogels, bioceramics, and biomimetic metals-with emphasis on their design principles, structural characteristics, and applications in disease detection, targeted therapy, and biomimetic implants. By examining natural models such as plant surfaces, animal tissues, and microbial systems, we discuss how these inspirations have led to materials with enhanced biocompatibility, responsiveness, and mechanical performance. Current challenges and future directions in the field are also addressed.

## 1 Introduction

Since the beginning of the 21st century, humanity has witnessed rapid technological advancements. The continuous evolution of electron microscopy and related imaging techniques has enabled researchers to extend their exploration of the natural world from the macroscopic to the microscopic scale. By mimicking the composition, structure, formation processes, and functions of organisms found in nature, researchers have designed and fabricated a wide variety of bio-inspired materials with significant potential for biomedical applications (Ran et al., 2022). This potential is exemplified by the work of Antonios G. Mikos, who developed a highly biomimetic aligned fibrin gel (AFG) scaffold (Chai et al., 2022). Upon transplantation into an injured cerebral cortex, this scaffold effectively promoted the migration, differentiation, and maturation of endogenous neural stem cells, ultimately leading to the restoration of neurological function. Beyond such direct therapeutic applications, these materials often integrate other advantageous traits derived from biological systems, including superior impact absorption (Wan et al., 2022), high-temperature resistance (Saleemi et al., 2022), self-repairing capabilities (Zhang et al., 2024), and biodegradability (Deng et al., 2023). These combined properties enable them to maintain stable performance even under extreme conditions, thereby expanding their application potential across diverse medical scenarios. Despite these remarkable advantages, the clinical translation of bio-inspired materials faces substantial hurdles. Challenges such as difficulties in miniaturization, structural complexity, low production yields, and high manufacturing costs impose economic burdens on their practical application. More critically, potential instabilities in both biocompatibility and mechanical properties raise concerns regarding long-term safety, necessitating further in-depth investigation.

In recent years, the field of bio-inspired materials has witnessed a proliferation of excellent reviews, each offering unique perspectives. For instance, Chen et al. (2022) provided a comprehensive overview of molecular imprinting techniques for creating bio-inspired recognition materials, while Zhang et al. (2022a) explored bioinspired functional surfaces tailored for medical devices. Building upon this body of literature, the present review aims to offer a distinct and integrated perspective by systematically examining and comparing three fundamental material categories: hydrogels, bioceramics, and biomimetic metals. Hydrogels, owing to their ability to mimic the soft, hydrated extracellular matrix, are particularly suitable for soft tissue engineering and drug delivery. Metals provide the essential load-bearing strength required for orthopedic and dental implants. Bioceramics exhibit superior bioactivity and osteoconductivity, making them ideal for bone regeneration applications. This review concisely outlines the historical development and structural characteristics of these three material classes, while also elucidating their applications in disease monitoring, therapeutic interventions, and the design and implementation of human implants.

We acknowledge the importance of hybrid materials, bioinspired polymers, and surface modification strategies; however, these topics are integrated into the discussion of the primary material categories. For example, polymer-ceramic composites are addressed within the bioceramics section, and bioinspired surface functionalization is covered in the section on biomimetic metals. Unlike existing reviews that often focus on a single material category or specific application domains, our approach elucidates the common bioinspired design principles shared by these materials, as well as their unique and complementary roles in addressing diverse biomedical challenges-ranging from soft tissue engineering and hard tissue regeneration to implantable medical devices.

## 2 Principles of bioinspiration in material design

### 2.1 Plants

Plants serve as a rich source of inspiration for material design, offering lessons that span multiple levels of biological complexity-from fundamental metabolic functions to sophisticated environmental response mechanisms. At the most fundamental level, plants demonstrate how to harness energy from the environment. Photosynthesis, the process by which leaves convert solar energy into chemical energy, has directly inspired the development of artificial photosynthetic systems, including bio-inspired solar cells and panels (Barber and Tran, 2013). This represents a fundamental functional biomimicry approach. The ability to efficiently capture and convert energy from the environment is a critical frontier for biomedicine. It inspires the development of self-powered implantable medical devices, such as pacemakers or neural stimulators, which could harvest energy through biocompatible photovoltaic or photocatalytic skin, thereby eliminating the need for repeated surgeries to replace depleted batteries. This principle is essential for the long-term functionality of future therapeutic implants and micro-robots. Moving beyond function to interfacial properties, the lotus leaf exemplifies how surface architecture can dictate material performance. Its remarkable self-cleaning property, arising from micro- and nano-scale hierarchical structures coupled with low-surface-energy wax crystals, has inspired the design of superhydrophobic surfaces that prevent biological fluid adhesion and reduce infection risk in medical devices (Liu et al., 2017).

**FIGURE 1:**
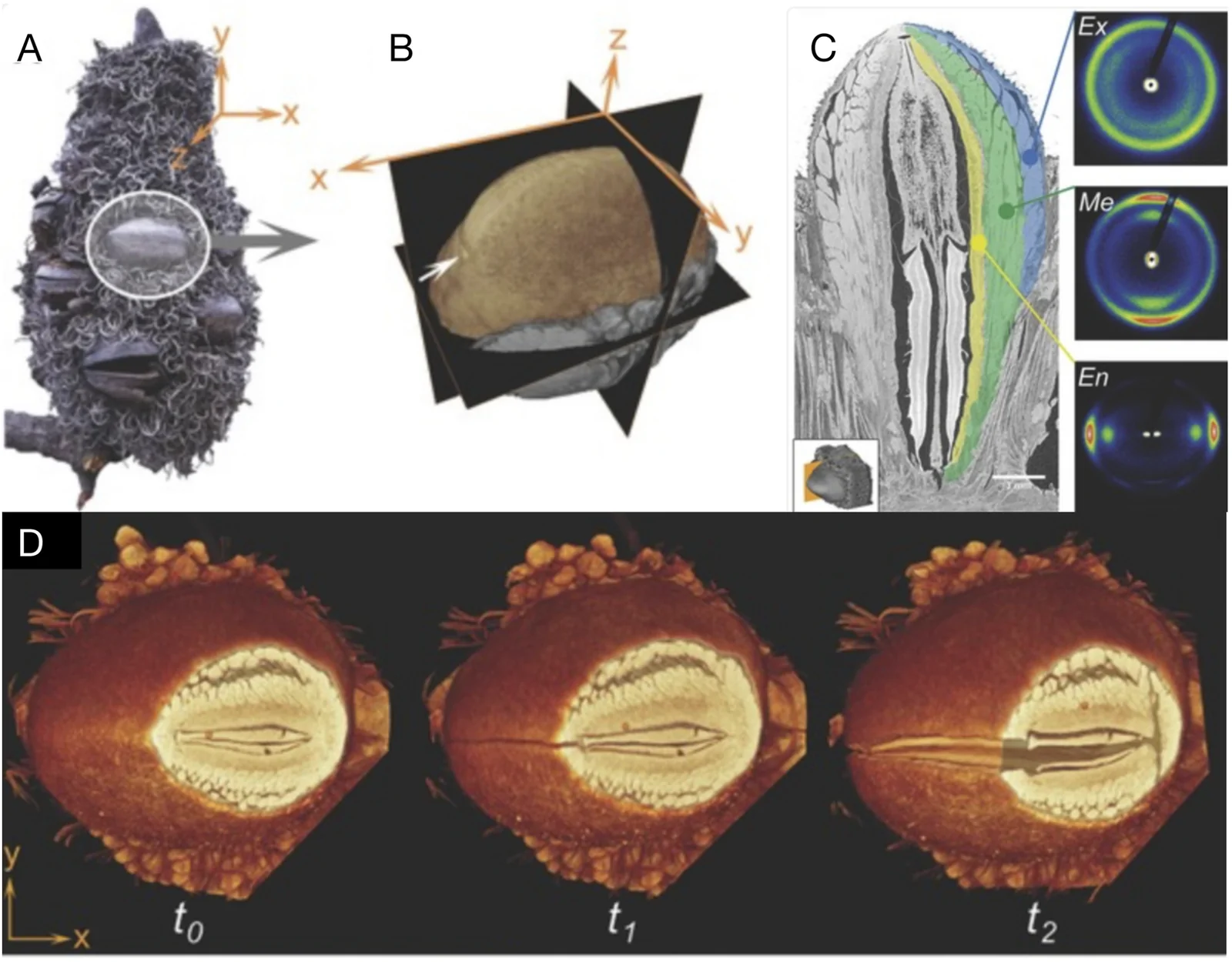
Banksia seed pod and its inspiration for smart drug delivery systems (Huss et al., 2018). **(A)** Spatial orientation of a **(B)**. attenuata follicle on the mature fruit cone. **(B)** Directions of the three cutting planes. **(C)** Internal structure and organization of **(B)**. attenuata follicle tissue. **(D)** Time-resolved opening and composition of specialized follicle tissue. t _0_: before heating; t _1_: after some minutes of heat exposure; t _2_:after a longer period of heating. This hierarchical, stimuli-responsive opening mechanism has inspired the design of smart drug delivery systems and soft robotic actuators that sense environmental cues and execute controlled release in response to specific triggers such as temperature, pH, or humidity.

At the highest complexity level, certain plants exhibit integrated sensing-actuation systems for environment-triggered responses. Banksia seed pods exemplify this: they store seeds dormant for up to 15 years, releasing them only after two distinct rainfall signals (Eder et al., 2018). This controlled release is enabled by the pod’s hierarchical architecture and compositional heterogeneity. When temperatures exceed 45 C, surface wax melts-sealing dry-induced micro-cracks-while the inner endocarp softens, priming rupture upon subsequent humidity increase. This process relies on coordinated action of cellulose, hemicellulose, lignin, wax, and tannin (Huss et al., 2018) (Figure 1). The Banksia pod thus provides a blueprint for soft robots and intelligent drug delivery systems that sense environmental cues and execute controlled release. These examples collectively delineate a progressive hierarchy in bio-inspired design. This progression underscores that materials must not only possess appropriate bulk properties but also exhibit smart, environment-responsive behaviors for applications such as targeted drug delivery and adaptive implants.

### 2.2 Animals

**FIGURE 2:**
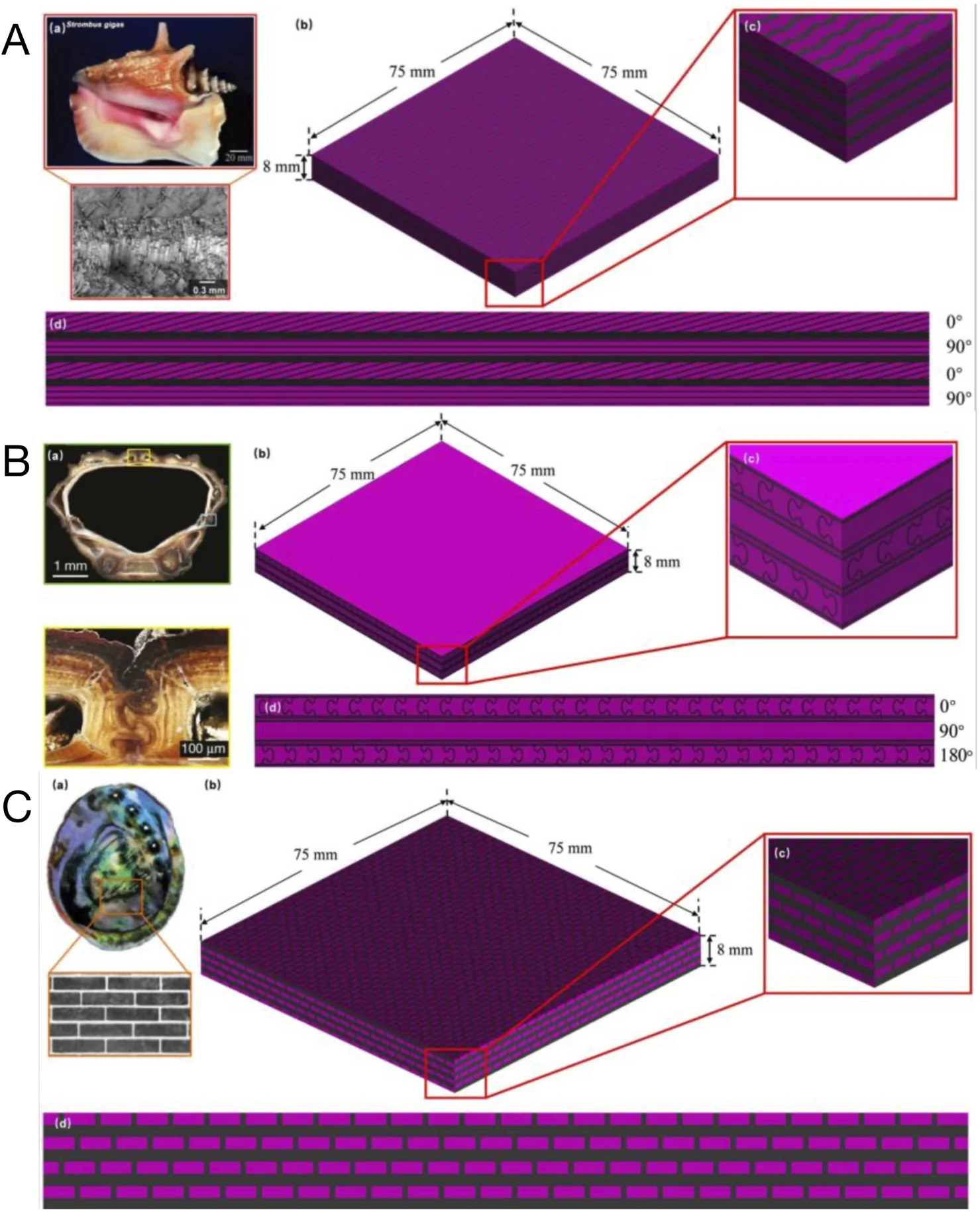
Biological structural motifs inspiring impact-resistant composite materials (Wan et al., 2022). **(A)** Bio-S: Conch Shell Mimetic Design: (a) Natural microstructure of the conch shell (Strombus gigas). (b) Schematic of the Bio-S specimen model. (c) Local architectural features of Bio-S. (d) Side view of the Bio-S layered structure. **(B)** Bio-B: Beetle Exoskeleton Mimetic Design: (a) Natural microstructure of the beetle exoskeleton. (b) Schematic of the Bio-B specimen model. (c) Local architectural features of Bio-B. (d) Side view of the Bio-B layered structure. **(C)** Bio-N: Nacre Mimetic Design: (a) Natural microstructure of nacre. (b) Schematic of the Bio-N specimen model. (c) Local architectural features of Bio-N. (d) Side view of the Bio-N layered structure. These biological architectures evolved for optimal impact resistance and energy dissipation, have inspired the design of synthetic composites for load-bearing orthopedic implants, protective coatings, and aerospace materials requiring high toughness and damage tolerance.

In addition to plants, animals provide a rich source of design principles. For mechanical resilience, nature offers numerous solutions for impact resistance and energy dissipation. The mantis shrimp, for instance, accelerates its dactyl club to 20 m/s within 3 m to shatter mollusk shells, generating enormous impact energy (Weaver et al., 2012). Similarly, bighorn sheep collide at speeds of 9 m/s without fracturing their horns (Huang et al., 2017), while insect and crustacean exoskeletons resist static extrusion and low-speed impact (Rivera et al., 2020). Biological teeth and bones further exemplify long-term durability, requiring exceptional wear and fatigue resistance over decades of use. These mechanical paradigms have inspired synthetic analogs. By mimicking the helicoidal fibre layup of the mantis shrimp exoskeleton, researchers demonstrated that controlling the printing direction in each layer can significantly enhance the out-of-plane strength and impact toughness of 3D-printed plates (Garg et al., 2023; Long et al., 2022). This material holds promise for bone tissue implants and aerospace applications. Similarly, 3D-printed bionic composite plates mimicking conch shells, beetle exoskeletons, and nacre were fabricated, and drop tower tests revealed that all three designs exhibited superior low-velocity impact resistance compared to monolithic plates (Wan et al., 2022) (Figure 2).

**FIGURE 3:**
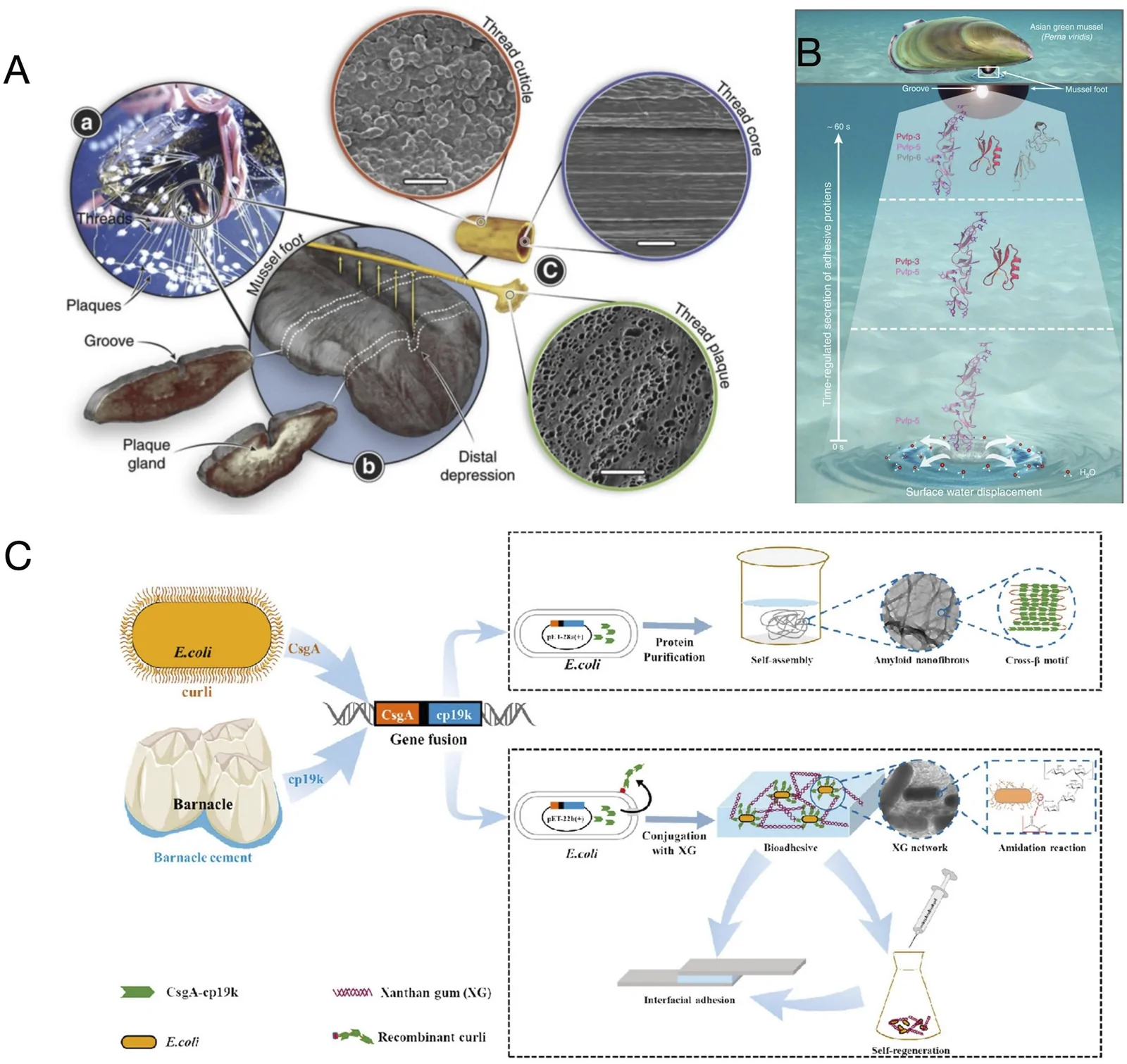
Marine organisms inspiring wet-adhesive biomaterials for tissue repair. **(A)** Morphology of mussel byssus. **(a)** Mussel secreting threads onto plexiglass via foot. (b) μ-CT of iodine-stained foot (ventral view), showing internal glands and groove. Threads form by protein secretion into ventral groove. (c) Single thread schematic with SEM images: cuticle (3 μm), fibrous core (5 μm), adhesive plaque (50 μm) (Priemel et al., 2017). **(B)** The adhesive secreted by Asian green mussels (Petrone et al., 2015). **(C)** Modular design of a barnacle-inspired living-cell adhesive. Drawing key inspiration from barnacle cement, the CsgA-cp19k fusion protein was designed by incorporating barnacle cement protein (cp19k) into the *E. coli* curli system (CsgA). The fused gene was cloned into pET vectors for protein purification and curli formation. Purified CsgA-cp19k self-assembles into amyloid nanofibers with strong adhesion. On cell surfaces, it forms a recombinant curli network. Combined with xanthan gum, engineered *E. coli* yields a self-regenerating living-cell bioadhesive (Li et al., 2022a). The protein chemistry and secretion mechanisms of mussel and barnacle adhesives—which enable strong, durable underwater attachment—have directly inspired the development of surgical tissue adhesives, wound sealants, and wet-surface coatings for biomedical implants, offering alternatives to sutures with reduced pain and no need for stitch removal.

In wet environments, barnacles and mussels inspire diverse adhesion strategies with significant clinical relevance for tissue adhesives, which offer advantages over sutures-reduced pain and no need for stitch removal (Petrone et al., 2015; Li et al., 2022a) (Figure 3). At the molecular level, Yahao Liu et al. designed a self-healing polyurethane integrating mussel-inspired catechol motifs with dynamic disulfide bonds (Liu et al., 2021). It achieves 98.4% self-healing efficiency in 6 h at ambient temperature and retains 98.1% efficiency after 2 h in 60 °C water, suitable for protective coatings and flexible sensors. Beyond chemistry, researchers mimicked the formation mechanism of mussel adhesives-liquid-liquid phase separation (LLPS) followed by maturation. Engineered proteins with mammalian low-complexity domains underwent LLPS, forming dense amyloid nanofiber coatings on diverse surfaces (Teflon to microfluidics) over wide pH (3-11) and salt ranges, exhibiting strong underwater adhesion (Cui et al., 2019). Addressing the clinical challenge that most adhesives fail in humid physiological environments, Lee et al. developed a polyallylamine-based adhesive with excellent wet adhesion and autonomous recovery, making it suitable for surgical wound closure (Lee et al., 2021). These examples illustrate a progression from chemical mimicry to mechanism replication and clinical translation.

### 2.3 Microorganisms

In addition to animals and plants, microorganisms have also provided abundant inspiration. Their motor capacities and responsiveness to external stimuli can be harnessed for the precise transport and assembly of drugs or genes. For example, the *Chlamydomonas reinhardtii* is a unicellular green alga with flagella, allowing it to move freely in a liquid environment. Drawing inspiration from this, Weibel et al. adsorbed drugs on their surface, and then precisely controlled the direction and speed of motion using specific wavelengths of light (Shchelik et al., 2021). This approach enabled the directional transport and release of the particulate cargo. Moreover, Martel et al. controlled the direction and speed of motion of *Magnetospirillum gryphiswaldense* using the external magnetic field, allowing it to carry the medicine and transport it exactly to the intended location (Felfoul et al., 2016). Furthermore, sperm cells are loaded into the magnetic microcap containing doxorubicin, enabling them to be magnetically guide and transport and release drugs together for the treatment of cervical and ovarian cancers (Xu et al., 2020).

**FIGURE 4:**
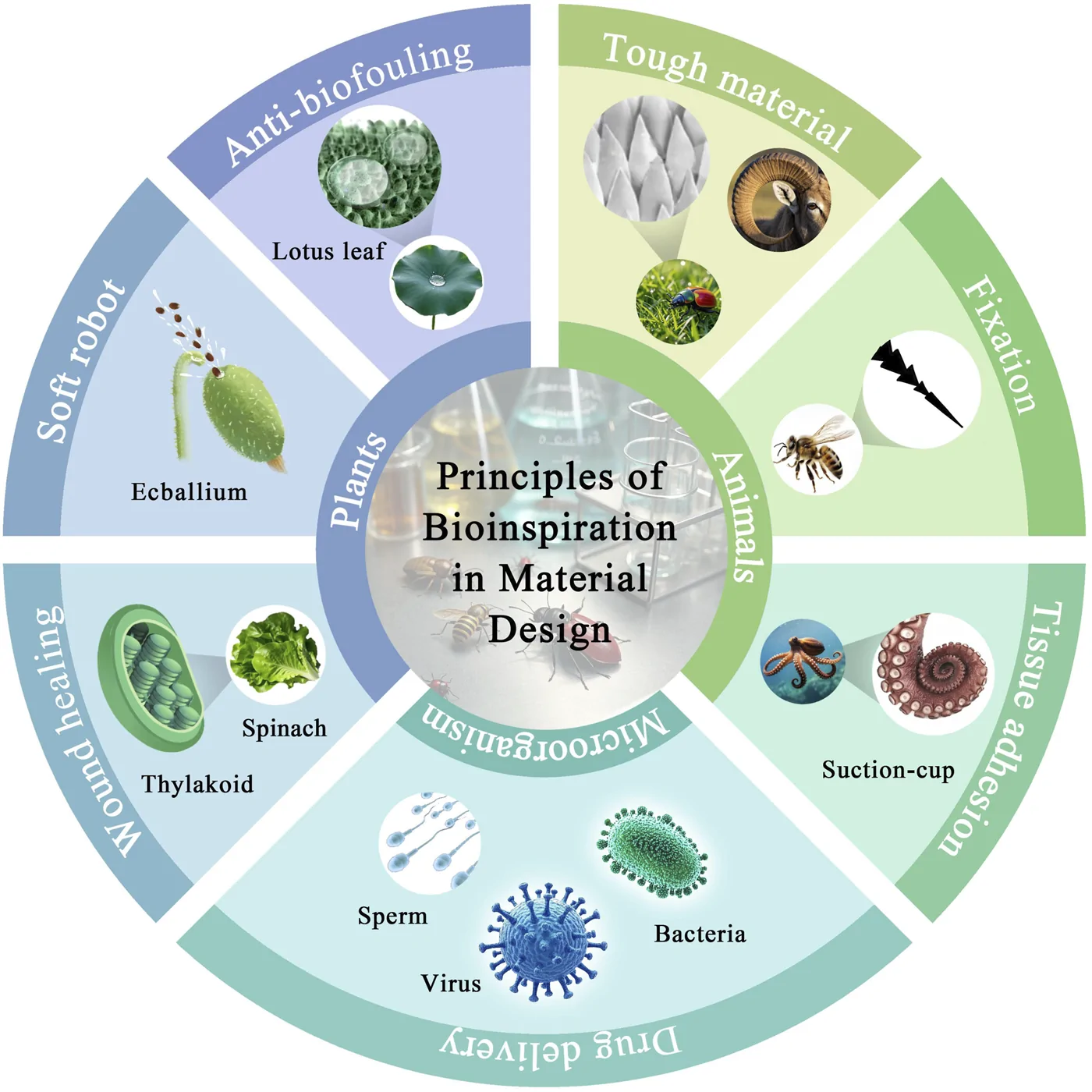
Principles of bioinspiration in material design.

As summarized in Figure 4, these examples from plants, animals, and microorganisms illustrate a progressive hierarchy in bio-inspired design. From fundamental energy conversion and surface engineering to complex environmental response systems, mechanical resilience, wet adhesion, and finally, precision delivery systems inspired by microorganisms. Together, these examples across different levels of biological complexity offer valuable inspiration for developing advanced biomedical materials.

## 3 Hydrogel

### 3.1 Historical development of hydrogels

The hydrogel is a polymer with a hydrophilic group, consisting of three-dimensional polymer network and plenty of water. Its network structure can absorb plenty of water to expand, and it is insoluble in water (Ho et al., 2022). The research and application of hydrogels have undergone many important developments. The history of hydrogels dates back to 1894 when the concept of “hydrogel” was first introduced to describe the colloidal gel. Later, DuPont scientists reported the synthesis of poly-2-hydroxyehtyl methotrexate hydrogel in 1936 (Das et al., 2021). In the 1950s, Otto Wichterle and Drahoslav first synthesized PHEMA (poly-2-hydroxyethyl methotrexate), a materials not only absorbs a lot of water, but also exhibits good biocompatibility. This is the first attempt to design polymers for human use that meet biocompatibility criteria and apply them to the production of contact lenses (Kopecek, 2009). At that time, scientists started to design and synthesize a large number of hydrogels, which became a hot spot, and explored the synthesis and properties of polymer materials. At the conclusion of the 20th century, scientists initiated research into smart hydrogels that possess the ability to respond to diverse environmental stimuli, such as temperature, pH values, light, and magnetic fields. These hydrogels are known as stimulus-responsive hydrogels, and their development has brought a revolutionized fields such as drug controlled release, smart sensors, flexible wearable devices and other fields. In 1992, Hoffman was the first to report a hydrogel capable of responding to multiple stimuli. The hydrogel is constituted by Nisopropyl acrylamide (NIPAM), acrylic acid and divinyl silicone rubber (Das et al., 2021). In the 21st century, the rapid progress in nanotechnology has given rise to the development of composite hydrogels, designed to fulfill contemporary requirements.

By combining hydrogels with nanometer materials, these composites gain enhanced functionality and capabilities. Novel functional properties are imparted to these hydrogels by embedding nanometer materials into the hydrogel matrix. For example, inspired by mucus secretion mechanisms, Feng et al. developed a novel magnetically responsive semi-convertible hydrogel (MSCH). Magnetically responsive gelatin and amino-modified Fe_3_O_4_ nanoparticles were embedded into the covalent network of PVA. Under the influence of a magnetic field, the triple helix structure of gelatin was disassembled and driven by the force of magnetic particles, while the covalent network maintained the integrity of the hydrogel structure and enabled it to switch between gel state and sol state (Gao et al., 2024). The material can be used for artificial joint implantation, soft robots and reducing friction and wear.

### 3.2 Classification of hydrogels

Based on their source, hydrogels can be classified into three categories: natural, semi-synthetic polymeric, and synthetic hydrogels. Natural hydrogels are obtained from various natural polymer substances, including hyaluronic acid, chitosan, alginate, and collagen (Yuan et al., 2023). Owing to its notable benefits in terms of biocompatibility and biodegradability, natural hydrogels demonstrate significant potential for applications within the biomedical domain. However, its widespread application is constrained by factors such as its inferior mechanical properties, limited stability, variability between batches, and the high degree of processing complexity. In future developments, the integration of 3D printing technology with other materials holds potential to significantly augment the properties of natural hydrogels and broaden their range of applications. Synthetic hydrogels are made from synthetic polymer materials that can be of customized design to meet specific requirements, offering controllable mechanical properties and chemical stability. Semi-Interpenetrating Polymer Networks (Semi-IPNs) involve the integration of an extra polymer, which may be uncross-linked or partially cross-linked, into a polymer network that has already undergone cross-linking. This structure provides semi-polymeric hydrogels with stability of cross-linked networks and the mobility of uncross-linked polymers to exhibit unique physical and chemical properties (Mehta et al., 2023). In the future development, hydrogels are anticipated to become increasingly intelligent, versatile, and eco-friendly. The application range of hydrogels will be further expanded to promote the progress in many fields (such as healthcare, environment and energy) through interdisciplinary research and technological innovation. Solving the current challenges in mechanical properties, stability and large-scale production will be an important direction of future research, too.

### 3.3 Crystallisation

Hydrogels can be classified into amorphous, crystalline, or semi-crystalline forms based on their structural characteristics. Amorphous hydrogel refers to a type of hydrogel where polymer chains are arranged in a disordered manner, without the formation of crystalline region within its network structure. In amorphous hydrogels, a three-dimensional network is generally established through either physical or chemical cross-linking among long-chain polymers. Due to their disordered structure, the hydrogel exhibits high flexibility and good water absorption (Ho et al., 2022). For example, Amir Erfani et al. mixed the antibody with sodium alginate solution to make an antibody with no fixed form. This method can not only maintain the stability of biological agents, but also provide convenient injection forms (Erfani et al., 2023). Crystalline hydrogel refers to a type of hydrogel where polymer chains arranged in a highly ordered manner, forming crystalline region in the network structure of hydrogel. The crystallization region is a regular structure formed by the polymer segments through strong interactions between molecules. This region is characterized by high hardness and strong stability (Matsuda et al., 1994; Shi et al., 2020; Zhang et al., 2014a). Semi-crystallin hydrogel feature both crystalline and amorphous regions within their network structure. The hardness and strength of the material are attributed to its crystalline regions, whereas the amorphous regions enhance its flexibility and facilitate water absorption. This partially crystalline structure allows semi-crystallin hydrogels to achieve a balance between mechanical properties and swelling properties. For example, Fengcai Lin et al. designed an ionic conductive hydrogel with a dual network structure composed of polyurea (PU) and polyvinyl alcohol (PVA), referred to as PU/PVA-Li hydrogel. When subjected to external pressure, the physical interactions between PU and PVA chains, such as intermolecular/intramolecular hydrogen bonds and π-π stacking are disrupted. As a result, the energy is absorbed and dissipated. The hydrogel’s integrity is preserved by the highly crosslinked PU network, preventing any cracks from forming. The hydrogel possesses not only exceptional strength but also remarkable toughness and resistance to fatigue (Lin et al., 2023).

### 3.4 Surface functional groups

The functional groups in hydrogels are specific chemical units linked to the polymer chains of the hydrogel’s polymer network. They not only determine its chemical properties of the hydrogel, but also have an important influence on its physical properties and biological properties. For example, functional groups can regulate its hydrophilicity (Table 1), degree of cross linking and mechanical strength to influence its performance in different environments. By carefully designing and selecting different functional groups, the structure and properties of hydrogels can be precisely tailored to meet the needs of specific applications. The hydrogel is of extensive application potential, such as in drug release, tissue engineering, wound dressing, environmental remediation and other fields based on such customization capabilities (Olad et al., 2018; Peng et al., 2012; Zain et al., 2018; Zamani and Taherzadeh, 2010).

**TABLE 1:** Functional groups on hydrogel surfaces and their functions and applications.

Functional groups	Characterizations	Related applications	References
-OH	Hydrophilic, creates hydrogen bonds with water molecules to preserve its moist state	Wound Dressing	Ho et al. (2022)
-COOH	Weakly Acidic, pH responsive, Strongly Ionic	Drug controlled release system Wound monitoring	Cheng et al. (2023)
-NH_2_	Weakly basic group, interact with other functional groups, forming amide or imine bonds	Fluorescent markers Disease detection	Mehta et al. (2023)
-SO_3_H	Hydrophilic, Absorbability	Catalytic, Water purification Hygroscopic materials, Adsorbent	Sajjadi et al. (2024)
-CHO	Biofunctionalization of modified hydrogels	Drug controlled release system Self-healing hydrogel	Li et al. (2024)

## 4 Bioceramics

### 4.1 History of bioceramics

Bioceramics are a class of ceramic materials employed within the biomedical domain for their excellent biocompatibility, durability, and mechanical properties. They are extensively employed in fields such as orthopedics, traumatology, dentistry, and tissue engineering. In 1969, Hench first introduced the concept of “Bioactivity,” which refers to the ability of a material to form chemical bonds with surrounding tissues in the body (Hench and Paschall, 1973). This concept, which revolutionized the development of bioceramics, prompted researchers to design a range of bioactive ceramic materials. Following this, Hench developed bioactive glass (Bioglass), which can directly bond with bone tissue in the body and is extensively used in bone repair and regeneration (Hench, 1988). This innovation represented a crucial milestone in the realm of bioceramics, propelling advancements in the field of biomedical materials. Subsequently, hydroxyapatite (HA) (George et al., 2022), bioactive ceramics (Wang et al., 2024), and zirconia (ZrO_2_) (Bannunah, 2023) were successively introduced. By the 1990 s, biodegradable ceramic materials, such as calcium phosphate ceramics (Navarro et al., 2004), and bioceramic composites (Sivakumar et al., 2023) had emerged. These materials combine the advantages of different materials, enhancing toughness and mechanical strength while maintaining good biocompatibility and degradability. Entering the 21st century, the development of bioceramics has entered a new phase, with research focusing on personalized medical applications, such as smart bioceramics and the integration of bioceramics with 3D printing technology.

### 4.2 Classification of bioceramics

Bioceramics can be categorized according to their interactions with biological tissues, their physicochemical characteristics, and the specific fields of application (Table 2). The main categories include inert bioceramics, bioactive ceramics, biodegradable ceramics, bioinert composite ceramics, smart bioceramics, and nanostructured bioceramics. These materials' unique properties, such as biocompatibility, bioactivity, and mechanical strength, make them essential components in implants and tissue engineering. They provide dependable solutions specifically for the medical domain, especially in the fields of orthopedics and dentistry. As technology advances, the application range of bioceramic materials will expand further, fostering the development of more advanced and personalized medical solutions.

**TABLE 2:** Classification of bioceramics and their applications and characteristics.

Category	Examples	Applications	References
Bioactive Ceramics	Hydroxyapatite	Bone repair, bone replacement, bone fillers, implants	Tanner (2010)
	Bioactive glass (BGs)	Tissue scaffolds, bone replacement, craniofacial and dental surgery	Fernandes et al. (2017)
	Tricalcium phosphate	Tissue replacement, periodontal tissue repair, oral-maxillofacial reconstruction, bone fillers, drug delivery systems	Dorozhkin (2010)
Bioinert Ceramics	Aluminum oxide	Artificial joints, dental implants, dental coatings	Keppler et al. (2020)
	Zirconium oxide (ZrO_2_)	Crown and bridge supports, coatings	Sethu et al. (2017)
	Silicon carbide ceramics (SiC)	Femoral head wear-resistant coatings, Sensing applications	Liu et al., 2024, Zhang (2022)
Biodegradable Ceramics	β-Tricalcium phosphate (β-TCP)	Bone fillers, bone repair, drug delivery	Huang et al. (2024), Wu et al. (2023)
	Dicalcium phosphate dehydrate (DCPD)	Bone fillers	Sheikh et al. (2015)
	magnesium phosphate ceramics (MgP)	Bone repair, Modular fabrication, metal protective coatings	Sarkar (2024)
Smart Bioceramics	Temperature-responsive ceramics	Liquid recovery, oil-water separation	Yin et al. (2022)
	Magnetic-responsive ceramics	Drug delivery systems	Zhang et al. (2022)

### 4.3 Microstructure of bioceramics

The microstructure of bioceramics, including factor such as porosity, particle size, and distribution, directly influences the material’s mechanical properties, bioactivity, and degradation behavior (Ginebra et al., 2018). The porous architecture of bioceramics is essential for their use in bone tissue engineering. Such structure not only promotes cell adhesion, proliferation, and differentiation but also facilitates the growth and infiltration of blood vessels and new bone tissue into the bioceramic, thereby enhancing the material’s biointegration. The pore size and connectivity affect the material’s degradation rate and bone integration capability. Ideal bioceramics typically have a highly interconnected porous structure, featuring pore diameters that span from 100 to 500 μm, to effectively support bone regeneration (Von Doernberg Et Al., 2006; Zhang et al., 2014b). Nanometer-scale particles can offer a larger surface area, promoting bioactivity. For example, CaP with a microporous structure exhibits higher osteogenic potential and even greater osteoinductive capacity,than its non-microporous counterpart (Barradas et al., 2010).

## 5 Biomimetic metals

### 5.1 History of biomimetic metals

Biomimetic Metals is an interdisciplinary process that combines materials science, engineering, biomedicine, and bionics. The research and application of biomimetic metals are primarily focused on developing metallic materials with properties similar to human tissues for biomedical implants, such as bone plates, bone screws, dental implants and artificial joints. The practice of employing biomimetic metals can be traced to the second century AD, when early European civilizations began implanting iron teeth into human skeletal structures (Su et al., 2018). Moving forward to the 1940 s, cobalt-chromium alloys, stainless steel and titanium began to be used as medical implants. However, traditional metallic materials have limitations, such as corrosion caused by the physiological environment, which can lead the diffusion of metal ions into surrounding tissues, and causes toxicity and side effects. Additionally, the difference in Young’s modulus causes the so-called “stress shielding,” which may result in implant loosening (Wu et al., 2017), thereby limiting their long-term application. As known of biomaterials and human tissue properties deepened, scientists began designing biomimetic metal materials by mimicking the structures and functions found in nature. The focus of research shifted towards enhancing biocompatibility by altering the surface properties of metallic materials. For instance, Implants made of titanium alloy often incorporate hydroxyapatite (HA) coatings on their surfaces to substantially enhance osseointegration (Lacefield, 1988). Entering the 21st century, the research and application of biomimetic metals have rapidly advanced, with new material technologies and biomimetic design concepts continually being introduced, enhancing the diversity and optimizing the performance of biomimetic metallic materials. This includes porous metal materials and magnesium alloys (Zhang et al., 2022b). The Young’s modulus of magnesium alloys (41–45 GPa) closely matches that of human bone (40–57 GPa), effectively mitigating the stress shielding effect (Yan et al., 2020). The development of modern biomimetic metals not only brings their mechanical properties closer to those of biological tissues but also makes progress in terms of intelligence and multifunctionality, including smart biomimetic metals, composite biomimetic metal materials, and 3D printing technology (Lv et al., 2021).

### 5.2 Surface characteristics of biomimetic metals and classification of biomimetic metals

Currently, biomimetic metals can be categorized into two groups based on their reactivity within the human body. The first category is non-degradable medical metallic materials, which primarily include medical stainless steel, titanium and titanium alloys, rare refractory and precious metals, as well as shape memory alloys. These biomimetic metals retain their structure and function in the body over the long term without being gradual degradation or absorption by the biological environment, making them ideal for use in various long-term implants and medical devices (Su et al., 2018). For example, the Paragon stent made of Ni-Ti memory alloy, is used to treat coronary artery disease (Bakhshi et al., 2011). The other category is degradable medical metallic materials, which mainly include medical magnesium and magnesium alloys, iron alloys, and zinc alloys (Zhang et al., 2022c). A notable example is the Lekton Magic stent, typically made of Mg, is conveyed to the location of the vascular injury via the bloodstream, where it expands the vessel and then degrades on its own, avoiding the need for a second surgery and the inability to perform CT scans (Li et al., 2022b).

Metal surface roughness refers to the irregularities of the microscopic morphology of the metal surface and is usually used to describe the smoothness or unevenness (Bosshardt et al., 2017). Surface roughness has a significantly affect on the biological performance of biomimetic metals. Technologies like laser surface texturing (LST) and 3D printing (3DP) have the capacity to modify the macroscopic surface properties of metals, whereas sandblasting and acid etching are capable of creating intricate surface details at the microscopic level.

Plasma spraying and anodizing processes can further modify surface morphology at the nanoscale (Elias and Meirelles, 2010; Feller et al., 2015; Nagasawa et al., 2016). Surface treatments designed to produce rough textures can improve the primary fixation and stability of implants. In contrast to smooth ones, they offer superior mechanical interlocking at the bone-implant interface, thereby enhancing their interaction. Additionally, the use of textured surfaces on implants can boost the interface area with the surrounding tissue, thereby accelerating tissue healing. For example, in dentistry, using nano-modified titanium alloys increases surface roughness (Souza et al., 2019). However, a rougher implant surface tends to accumulate bacterial plaque more readily, thereby elevating the risk of peri-implantitis. Therefore, surface roughness must be achieved between enhancing biological activity and mitigating plaque accumulation (Lang and Jepsen, 2009).

Surface wettability, quantified by contact angle, critically governs the interfacial interactions between metallic biomaterials and the biological environment (Huhtamäki et al., 2018). A low contact angle indicates hydrophilicity (promoting spreading), while a high angle indicates hydrophobicity (repelling liquids). Wettability profoundly influences cell adhesion, protein adsorption, and blood compatibility, key determinants of implant performance (Feller et al., 2015). However, optimal wettability is application-dependent: hydrophilic surfaces enhance osseointegration for orthopedic implants, whereas hydrophobic surfaces minimize bacterial adhesion and infection risk (Valiei et al., 2022). To address these competing needs, wettability can be tailored through coatings, chemical treatments, or nanostructuring. A notable example is the SLActive titanium surface, which achieves superhydrophilicity via controlled chemical treatment, significantly improving implant-bone bonding efficiency (Souza et al., 2019). This demonstrates how fundamental wettability-biology insights translate into clinically optimized surface modifications.

To address diverse clinical needs, biomimetic functionalization techniques, such as surface coatings, nanostructuring, and chemical modifications, endow metallic materials with specific functions like cell adhesion, antibacterial properties, and tissue regeneration. Drawing inspiration from the lotus leaf, the study of superhydrophobic surfaces has been widely pursued for applications in engineering and biomedicine, due to their capability to decrease protein adsorption and prevent platelet adhesion and activation (Barthlott and Neinhuis, 1997). Researchers fabricated a superhydrophobic titanium surface using femtosecond laser ablation technology, effectively preventing bacterial adhesion (Aceti et al., 2022). The nanopatterned structure found on cicada wings exerts physical forces on bacteria, resulting in the disruption of bacterial cells. This mechanism was suggested by researchers Ivanova et al. (2013) and Pogodin et al. (2013), who later drew inspiration from it to produce titanium dioxide nanowire arrays on titanium implants, to regulate their antibacterial activity and osseointegration functions (Ivanova et al., 2013; Pogodin et al., 2013).

## 6 Bio-inspired materials for biomedical diagnosis and therapy

### 6.1 Disease detection

Disease detection can identify abnormalities at early stages, allowing for timely interventions that effectively reduce disease severity, transmission risk, and mortality rates. Therefore, a robust monitoring system is essential. Traditional methods of disease detection include laboratory testing, imaging, clinical manifestations and medical history. With advancements in biomaterials research, the emergence of bio-inspired materials offers more sensitive, real-time, and personalized tools for disease monitoring, driving progress in medical diagnostics and public health management.

Compared to bulk materials, nanomaterials possess a higher surface-area-to-volume ratio, making them increasingly adopted in various nanotechnological and nanomedical applications. For example, the detrimental impacts of ultraviolet (UV) radiation on human skin are widely acknowledged, highlighting the importance of accurate UV level detection. In recent years, spectroscopy based on surface-enhanced Raman scattering (SERS) has received extensive acclaim in the field of biosensing due to its outstanding sensitivity and selectivity. However, current SERS-based detection systems still face challenges in accurately detecting low and excessive levels of ultraviolet (UV). To overcome this challenge, designing nanostructures with high sensitivity and a large surface area is crucial (Park et al., 2023). Fabrication of nanostructures with numerous nanogaps and the formation of SERS substrates capable of accommodating high concentrations of analytes with a larger surface area are vital to addressing these limitations. In this study, scientists took inspiration from the morphology of Asterias forbesi (commonly known as the Forbes sea star) to enhance the performance of SERS substrates. The body and legs of the Forbes sea star have numerous protrusions, resembling structures that maximize the SERS effect by creating nanoscale hotspots. To replicate this structure, scientists enhanced the SERS effect by fabricating small-scale gold nanoparticles (GNPs) within a gold-silver bimetallic structure framework, mimicking the legs and protrusions of Asterias forbesi. Additionally, nanoparticles of iron oxide at the nanoscale level possess an intrinsic peroxidase-like activity, which is akin to that of natural enzymes, enabling their application in the oxidation of organic substrates for wastewater treatment or as a detection instrument. Based on this discovery, researchers devised an innovative immunoassay technique, utilizing antibody-modified magnetite nanoparticles that fulfill three roles: capturing, isolating, and identifying (Gao et al., 2007). These nanoparticles exhibit versatility, simplicity in production, and robustness, making them powerful tools with extensive potential applications in the fields of medicine, biotechnology, and environmental chemistry. Vitamin D has been identified as an important biological marker for diverse ailments, including rheumatoid arthritis, cancer, and cardiovascular diseases. Nonetheless, the sensitivity of vitamin D detection still requires enhancement. The villi of the small intestine increase the surface area of the intestinal wall, facilitating particularly efficient absorption of nutrients and enhancing digestive secretions. Drawing inspiration from the architecture of villi, researchers have crafted a biomimetic sandwich-type surface-enhanced Raman scattering (SERS) aptamer sensor utilizing silver nanovilli (AgNVs) for the exceptionally sensitive and selective detection of 25-hydroxyvitamin D3 (Kim et al., 2021). The densely packed nanovilli significantly enhances the Raman signal, making detection limit of this sensor being ten times lower than that of conventional methods such as plasmon-induced resonance and electroanalytical sensing. Inspired by the natural biosynthesis of complex minerals, researchers have also developed novel sensors with higher sensitivity, greater selectivity, and improved biocompatibility. These biominerals include silica found in diatoms and sponges, calcium carbonate shells in mollusks and coccolithophores, magnetosomes in magnetotactic bacteria, and hydroxyapatite in bones and teeth. These biominerals possess distinctive physicochemical characteristics, offering considerable capacity for biosensing applications. Diatom shells, which are species-specific biosilica structures with intricate geometric patterns, exhibit significant potential for biosensor applications due to their complex nanostructured morphology, high surface area, and species-specific architecture. Additionally, the porous nature of diatom biosilica allows the effective immobilization of biomolecules such as enzymes, antibodies, and DNA probes, thereby enhancing the sensitivity and specificity of biosensor devices. These natural structures can significantly amplify the surface plasmon resonance of self-assembled silver nanoparticles (NPs) deposited on their surfaces, functioning as optical amplification lattices. This amplification significantly enhances the sensor’s sensitivity in SERS, an efficient approach for molecular discovery based on unique light interactions. Initially, researchers coated clean diatom frustules (Pinnularia sp.) on glass slides. Next, the frustules were amino-modified to promote strong electrostatic interactions with subsequently adsorbed silver nanoparticles (AgNPs). Subsequently, goat anti-mouse IgG antibodies were selectively bound to the silver NPs. Finally, bovine serum albumin (BSA) was used to seal off non-specific attachment points. The introduction of gold plasmonic nanoparticles co-labeled with antibodies and signal-enhancing molecules like DTNB further amplified the SERS signal. The designed sensor demonstrated high selectivity for the analyte, binding exclusively with the target molecule (mouse IgG), and exhibited sensitivity exceeding that of conventional SERS sensors by 100-fold (Abdelhamid et al., 2024). Squire et al. later employed the same diatoms in fluorescent imaging immunoassays to detect cardiovascular disease biomarkers (Squire et al., 2019). The structural design of viral capsids also offers substantial inspiration for creating nanomaterials. Plant viruses, which are non-infectious to humans, typically exhibit complete uniformity in composition and size. For instance, the Tobacco Mosaic Virus (TMV), a helix-shaped viral particle exhibiting helical symmetry, is a virus with a single positive-polarity strand of RNA that contaminates tobacco and other solanaceous plants (Bruckman et al., 2014). The TMV capsid forms a molecular nanoscale cylinder with specified amino acid sites on its external surface. Steinmetz and colleagues created a dual-modal optical and MRI probe based on TMV, specifically targeting VCAM-1 (vascular cell adhesion molecule-1) receptors within atherosclerotic deposits within an ApoE^−/−^ mouse model. Chemically altered TMV, carrying Cy5, chelated Gd ions, and near-infrared dyes, was employed for focused visualization, demonstrating excellent visualization performance and good biocompatibility. This experiment demonstrated that by aiming at the cellular molecular receptors located on stimulated endothelial cells, large and effective payloads could be transported to areas of atherosclerotic plaque formation.

In addition to nanostructures, the multi-chamber structure of cells has inspired for the design of novel bioinspired materials. For example, molecular detection of HIV is crucial for the early diagnosis of HIV patients and for antiretroviral therapy, yet achieving simple and sensitive molecular detection remains a challenge. Inspired by the multi-chamber structure of living cells, researchers have proposed an artificial cascaded reaction system utilizing nanoporous membrane separation to enhance the compatibility of CRISPR-mediated multi-enzyme reactions (Li et al., 2023). By integrating this separated cascade reaction system with a microfluidic platform and glucose biosensing technology, a bioinspired, CRISPR-mediated cascade reaction (CRISPR-MCR) biosensor has been developed for HIV molecular detection. This system has achieved a detection sensitivity of 43 copies of HIV DNA plasmid and 200 copies of HIV RNA. Furthermore, scientists have derived inspiration from parasites. In 2024, Shahar Bracha utilized rods and dense particles of *Toxoplasma gondii* to deliver MeCP2 to neurons in the mouse brain, successfully breaching the blood-brain barrier (Bracha et al., 2024).

Pathogen-inspired carriers enable precise drug delivery, but safety must be engineered into their design. Taking *Toxoplasma gondii* as an example, attenuated strains are used to prevent uncontrolled proliferation, and therapeutic proteins are designed for release only within target cells (Bracha et al., 2024). Similarly, viral vectors such as adeno-associated virus (AAV) face challenges including immunogenicity and risks of genome integration. To address this, peptide-mediated delivery systems like HIV-TAT derivatives allow transient protein introduction without viral components, reducing long-term off-target effects (Allen et al., 2022). Bacterial carriers incorporate multiple safety features, such as dependence on nutrients not found in nature and the incorporation of suicide switches that trigger self-destruction under specific conditions (Choi et al., 2025). Immune responses must also be carefully managed; for instance, *Listeria monocytogenes* effectors (LLO, ActA) coated on synthetic cargoes can induce actin-driven expulsion from macrophages, minimizing lysosomal degradation and cytotoxicity (Van Staden et al., 2024). Finally, rigorous *in vitro* and preclinical studies are essential to establish drug safety and appropriate dosing regimens (Audouard et al., 2021; Eberhard et al., 2025).

### 6.2 Wound monitoring

Chronic wounds that are difficult to heal present a substantial biological, psychological, social, and financial strain on individuals and the healthcare infrastructure (Zhao et al., 2016). Currently, commonly used clinical wound dressings include bandages (Mohandas et al., 2019), foam sponges (Alven and Aderibigbe, 2020), and alginate dressings (Cressman et al., 1988). However, these traditional materials have several limitations. First, they often lack sufficient breathability, hindering the provision of an ideal oxygen exchange environment, which can impede wound healing. Second, changing these dressings can cause intense pain for the patient and may tear newly formed tissues, leading to secondary injury. Additionally, these dressings are limited in their ability to prevent microbial infections, leaving the wound vulnerable to bacterial invasion and potentially triggering allergic reactions, which can further exacerbate the condition. Most importantly, these materials typically struggle to maintain the necessary moist environment for the optimal, thereby delaying the healing process. Therefore, there is a need to develop more effective and targeted dressings to address the complex demands of chronic wounds (Alven and Aderibigbe, 2020). Hydrogel dressings, due to their moisture retention and adhesion properties, can address these issues and are widely used in wound care. However, traditional hydrogel dressings still require daily changes for visual wound assessment. Inspired by nature, researchers have engineered hydrogels that respond to stimuli. For example, receptors in human skin convert external stimuli into electrical signals, mimosa plants fold their leaves in response to touch, apple skins curl spontaneously as that dry, the “transpiration-guttation” phenomenon in plants, and the color-changing behaviors of chameleons and octopuses for self-protection (Su et al., 2015). These innovative hydrogel dressings can be combined with flexible wearable materials to respond to environmental factors such as pH, temperature, or color, thereby enabling real-time monitoring of the wound (Jin et al., 2024).

PH-responsive hydrogels are a category of stimulus-responsive system that react to changes in wound pH. Normal skin has a pH ranging typically from 4 to 6. When the skin is damaged, the pH initially decreases slightly and then rises, eventually returning to the normal range (5.5-6.5). However, in chronic wounds with inflammation, the pH remains in the alkaline range. Based on this principle82, Yuange Zong and colleagues have attached a near-infrared (NIR) fluorescent probe to the hydrogel network structure via an amide bond, forming an NIR fluorescent PIL-CS hydrogel. The NIR imaging device measures the fluorescence intensity of the hydrogel, providing a direct response to the pH at the wound site (Zong et al., 2023). Additionally, phenol red (a pH indicator) and two glucose-sensitive enzymes (GOx and HRP) were encapsulated in a zwitterionic polycarboxybetaine (PCB) hydrogel dressing, which can detect both pH and glucose levels (Zhao et al., 2020). Additional progress in this area pertains to hydrogels that are responsive to glucose. These hydrogels encompass hydrogels containing glucose oxidase (GOX), hydrogels with Concanavalin A (Con A) systems, and systems with phenylboronic acid groups (PBA). These glucose-responsive hydrogels are employed for the regulated release of therapeutic agents such as insulin in reaction to alterations in glucose levels (Chen et al., 2023). Temperature also plays a significant part in wound monitoring. For example, Qian Pang and colleagues developed a dual-network, temperature-responsive ionic conductive hydrogel with excellent stretchability, fast temperature responsiveness, and good conductivity. This was achieved by introducing a polyvinylpyrrolidone (PVP)/tannic acid (TA)/Fe^3+^ cross-linked network into the N,N-methylene bisacrylamide (MBAA) cross-linked poly (N-isopropylacrylamide-co-acrylamide) (P(NIPAAm-co-AM)) network (Pang et al., 2022). The hydrogel’s conductivity responds to environmental temperature changes, making it useful for temperature monitoring in wounds. Furthermore, Pang and his team created a novel hydrogel with a dual-layer design: the top layer is composed of flexible electronic components, which are encased in polydimethylsiloxane and feature an integrated temperature sensor as well as ultraviolet (UV) light-emitting diodes. An antibacterial hydrogel responsive to UV radiation comprises the lower layer. Temperature data gathered by the embedded sensor is then transmitted wirelessly to a smartphone via Bluetooth (Pang et al., 2020). These responsive hydrogel dressings, combined with embedded circuits and wireless communication technology, allow real-time monitoring of the wound microenvironment, furnishing medical practitioners with critical wound data to assess healing progress.

Furthermore, inspired by the feathers of hummingbirds and the exoskeleton of weevils (Fu et al., 2018), researchers have applied structural color to wound monitoring, utilizing its sensitivity to external environmental changes to alter the microstructure of materials. This approach facilitates non-invasive, real-time tracking of wound healing processes. For example, Wang’s team proposed a structural color ionic hydrogel, which is made from a polyacrylamide-poly (vinyl alcohol)-polyethyleneimine-lithium chloride (PAM-PVA-PEI-LiCl) inverse opal scaffold, combined with vascular endothelial growth factor (VEGF)-mixed methacrylated gelatin (GelMA) hydrogel on the surface. This material exhibits different structural colors under varying pressures and degrees of extension. The team then combined electrical stimulation (ES) with the structural color ionic hydrogel patch (SCIHP) for monitoring wounds in diabetic mice, finding that as the duration of ES increased, the SCIHP changed from red to green, possibly related to the temperature increase in SCIHP caused by the applied voltage. Additionally, Wang incorporated polymers of N-isopropylacrylamide (NIPAM) and acrylic acid (SA) into the synthetic inverse opal structure. This hydrogel dressing demonstrated shape memory effects during heating and cooling, allowing for reversible shape changes, with corresponding changes in structural color. Near-infrared irradiation enabled remote control of the hydrogel dressing’s shape and structural color changes (Wang et al., 2022). Moreover, a dressing was developed by combining polyacrylamide (PAM), silk fibroin (SF), poly (3,4-ethylenedioxythiophene)-poly (styrenesulfonate) (PEDOT, PP), and graphene oxide (GO). This dressing can closely adhere to the human body, simulating the sensory function of the skin, and displays significant color changes during stretching and bending, transmitting information to electronic apparatus for processing and feedback (Zhang et al., 2021). In addition, Bin and colleagues, inspired by the wet adhesion of spider webs and the structural color phenomena in chameleons, developed a self-reporting bioadhesive patch. This dressing not only demonstrates strong adhesion but also enables monitoring of its adhesiveness and the healing process through changes in structural color (Kong et al., 2022). Besides temperature, pH, and glucose, local levels of lactate and uric acid may be promising candidates for future research. Future work could improve smart dressings by increasing the diversity of monitoring indicators, refining algorithms for clinical wound outcomes, developing smarter medical plans for patients, and performing data analysis to examine the severity and distribution patterns of acute and chronic wounds.

### 6.3 Disease treatment

#### 6.3.1 Targeted therapy

Targeted therapy is a precise medical approach aimed at targeted eliminating of cancerous cells or specific diseased cells by identifying and attacking particular molecular markers or pathways while minimizing damage to normal cells (Saeed et al., 2023). Drug delivery technologies based on bioinspired materials are developed by mimicking the structures and functions found in nature, creating carriers capable of efficiently delivering drugs. These carriers leverage the intelligent characteristics of biological systems, such as targeting and responsiveness, to accurately identify diseased tissues and release drugs upon reaching the target location, thereby achieving therapeutic effects. Compared to traditional drug delivery systems, bioinspired material-based drug delivery technologies not only improve drug delivery efficiency but also significantly reduce systemic side effects, making treatments more precise and effective. They are widely applied in the treatment of cancers, infectious diseases, and chronic conditions (Karimi et al., 2016).

#### 6.3.2 Biomimetic pathogen delivery systems

Viruses serve as natural carriers of genes, capable of transferring biological information across diverse species. Viral drug delivery utilizes the natural infection mechanisms of viruses, with their pathogenicity removed through genetic engineering, and therapeutic drugs or genes are loaded into them (Karimi et al., 2016; Petrov et al., 2022). Once the virus enters the target cells, it can release the carried drugs or genes to exert therapeutic effects (Karimi et al., 2016). Inspired by this, Dong X et al. assembled silver nanoparticles onto the capsid proteins of M13 bacteriophages, utilizing the targeting function of the phages and the antibacterial effects of the nanoparticles, overcoming the limitations of traditional antibiotic treatments. This system precisely regulates the gut microbiota and remodels the tumor immune microenvironment, thereby inhibiting the growth of colorectal cancer (Dong et al., 2020). Additionally, Yoon Jung Hwang et al. constructed an engineered T7 bacteriophage that, by loading specific peptides, targets murine melanoma cells and carries a gene expressing granulocyte-macrophage colony-stimulating factor (GM-CSF) to achieve targeted therapy. The findings showed that this engineered phage effectively suppressed tumor progression in animal models (Hwang and Myung, 2020). Not only can bacteriophages serve as excellent carriers, but bacteria can also be genetically engineered to achieve targeted drug delivery, showing great potential in treating diseases such as cancer. Bacteria exhibit characteristics such as hypoxia tolerance, chemotaxis, and high permeability, and their flagella and chemotaxis receptors can target hypoxic tumor regions (Wang et al., 2023). Examples include reported bifidobacteria (Li et al., 2022c), *E. coli* (Yang et al., 2024), and *Salmonella typhi* (Wall et al., 2010). Additionally, magnetotactic bacteria containing magnetite or greigite-based magnetosomes can target hypoxic and necrotic tumor areas for targeted drug delivery. For instance, magnetotactic bacteria modified with nanoliposomes, guided by an external magnetic field, were able to deliver 55% of the drugs to hypoxic regions of colorectal cancer (Felfoul et al., 2016; Taherkhani et al., 2014). While pathogen-based drug delivery has strong targeting ability and high delivery efficiency, it also presents some limitations, including potential immunogenicity, possible host immune responses, genomic instability, and limited survival time *in vivo*. Moreover, the complex preparation and purification processes may affect its feasibility and safety in clinical applications (Karimi et al., 2016).

#### 6.3.3 Biomimetic cell membrane nanodelivery systems

Exogenous drugs often face limitations such as triggering immune responses that lead to inflammation or rejection, causing tissue incompatibility or damage, which can affect efficacy and safety. They may also be rapidly cleared or metabolized, shortening their half-life in the body and thereby impacting therapeutic effectiveness. Additionally, it can be challenging for these drugs to precisely reach target tissues or cells, leading to reduced efficacy. Biomimetic cell membrane nanodelivery systems involve isolating the cytoplasm and cell membrane of specific cells (such as erythrocytes, platelets, stem cells, and cancer cells) and encapsulating drug nanoparticles (such as polymer nanoparticles, liposomes, or metal nanoparticles) within the separated cell membrane to form a core-shell structure. This structure retains the cell membrane’s structure and functions while encapsulating the drug within the membrane to protect its activity (Li et al., 2019; Liao et al., 2024; Raza et al., 2024). Inspired by this, Diana Dehaini and colleagues fused red blood cell membranes with platelet membranes and coated them onto poly (lactic-co-glycolic acid) (PLGA) nanoparticles, termed [RBC-P]NPs. This hybrid membrane coating technology not only enhances stability and biocompatibility but also imparts specific functions of red blood cells and platelets to the nanoparticles, such as acetylcholinesterase activity and the ability to target tumor cells (Dehaini et al., 2017). Additionally, surface proteins on cancer cell membranes can specifically interact with cells at tumor sites, achieving efficient homologous targeting, concentrating the medication specifically at the tumor area and lessening its influence on surrounding healthy tissues.

For example, Zou and colleagues designed a delivery system based on glioblastoma, where they fused glioblastoma cell membranes with mitochondrial membranes to simultaneously recognize tumors and mitochondria, extending the circulation half-life of Gboxin from 0.47 h to 4.90 h (Lu et al., 2022; Zou et al., 2023). However, the preparation process for biomimetic cancer cell membrane drug delivery systems is relatively complex, and their safety still needs to be evaluated in clinical applications. Moreover, Xu and colleagues developed a dopamine-based biomimetic nanodelivery system that leverages the anti-ROS (reactive oxygen species) properties of platelet membranes. This system can accurately target brain hemorrhage sites, mimicking the natural hemostatic function of platelets and repairing damaged blood vessels (Xu et al., 2023).

### 6.4 Biomimetic implants

#### 6.4.1 Bone

**FIGURE 5:**
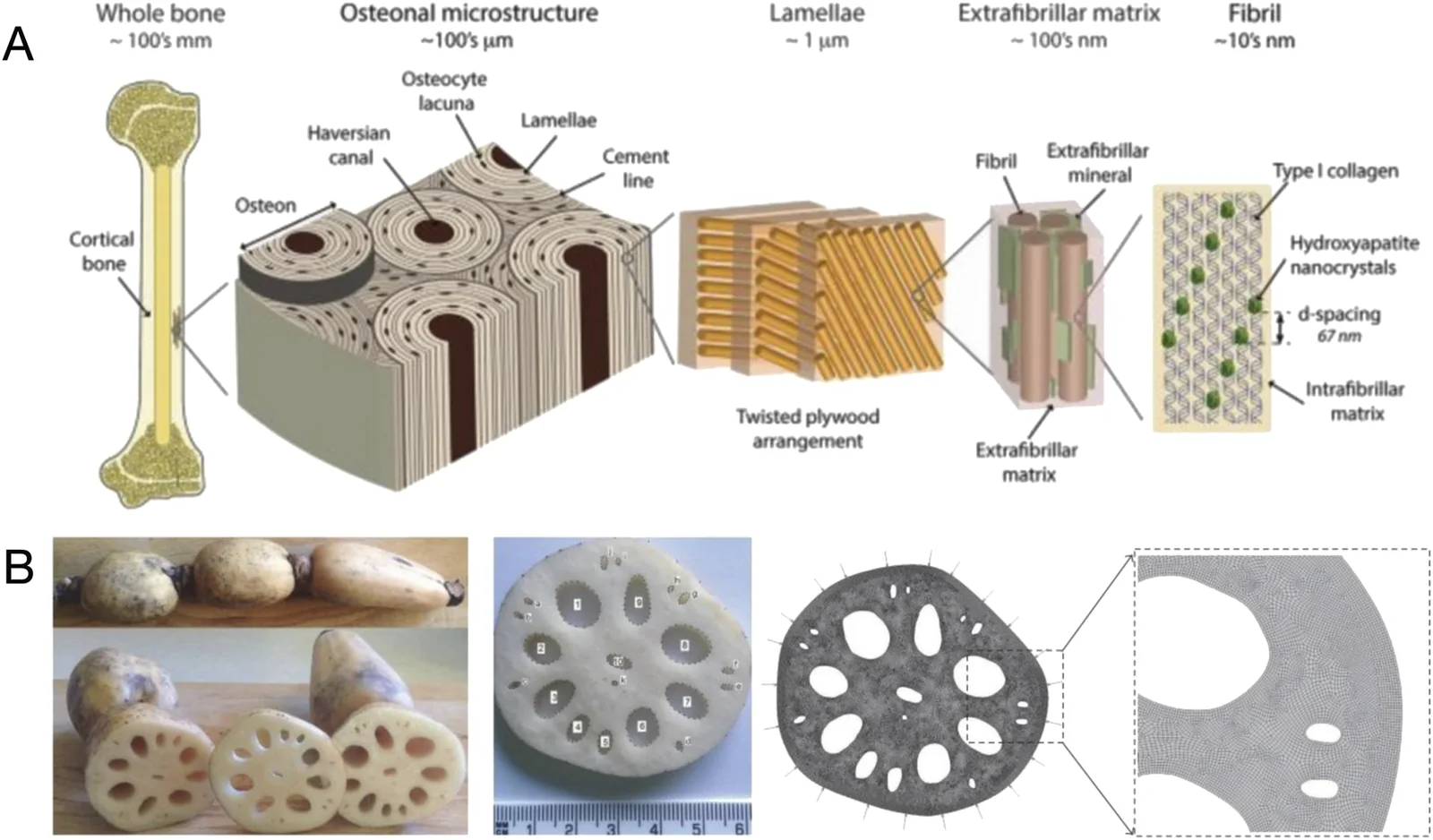
**(A)** Structure of human cortical bone (Zimmermann et al., 2016). **(B)** Structure of lotus root (Barrett et al., 1990).

Bone is a robust structure found in the bodies of vertebrates, primarily composed of 70% calcium phosphate and 30% collagen (Figure 5). It not only exhibits exceptional mechanical properties but also serves as a reservoir for calcium, phosphorus, and other ions within the body. Bone tissue is categorized into two primary types: cortical bone and cancellous bone. The porosity of cortical bone typically falls within a range of 5%–15%, whereas the porosity of cancellous bone can be significantly higher, reaching values of 40%–95%. These characteristics contribute to the anisotropic and complex layered structure of bones, enhancing their strength and toughness (Meng et al., 2023a). When bone tissue is taken out surgically because of trauma, infection, tumors, or developmental issues, it can cause significant bone defects. The primary treatment options include autologous bone grafting, allogeneic bone grafting, and the implantation of artificial bone repair materials. Autologous and allogeneic bone grafts face challenges such as limited donor availability and immune rejection. Consequently, bio-inspired artificial bone materials have emerged as viable alternatives (Munir et al., 2021; Yu et al., 2022). In designing artificial bone materials, it is essential to emulate the layered configuration and composition of natural bone. For example, hydroxyapatite (HA), which resembles the composition of bone, can be coated onto the surface of titanium alloy implants to enhance osseointegration capabilities (Lacefield, 1988).

Inspired by the adhesion mechanisms of mussels in natural systems, nano-hydroxyapatite was spontaneously modified onto porous PEEK through solution immersion, resulting in the formation of PEEK-PHA. This scaffold not only exhibits bone integration capabilities, owing to the characteristics of the polydopamine coating, PEEK-PHA but also can spontaneously assimilate lysozyme (Lys). The results of antibacterial test showed that the antibacterial rates of *Escherichia coli* and *Staphylococcus aureus* were 96.1% and 98.7%, respectively (Yu et al., 2022). Furthermore, inspired by the layered structure of nacre, Huijun Sun and colleagues developed a hydroxyapatite (HA)/polymer composite that mimics the microstructure of cortical bone using a bidirectional freeze-casting method. In this design, HA acts as rigid “bricks” embedded within a flexible polymer “mortar,” creating a microstructure similar to a brick wall. This layered arrangement effectively dissipates stress, enhancing the fracture resistance of the material (Tabrizian et al., 2023). Moreover, inspired by the porous internal structure of lotus roots, a biomimetic lotus root AKT bioceramic scaffold was constructed using 3D printing, with a porosity of up to 80% and mechanical strength exceeding 30 MPa. This scaffold can meet the requirements for non-load-bearing bone tissue repair. The parallel multi-channel structure facilitates nutrient transfer and material exchange between the scaffold and the bone defect microenvironment, significantly promoting bone tissue regeneration within the scaffold (Zhu et al., 2016).

In addition to these, the hinge of bivalves can withstand hundreds of thousands of repetitive opening and closing motions during their lifetime. In light of this, academician Yu Shuhong et al. investigated the layered design of mineralized tissues in the hinge of the bivalve pleated crown mussel to make the tissues deformable and fatigue-resistant. It is anticipated to be utilized for artificial implants (Meng et al., 2023b).

#### 6.4.2 Heart

As the origin of life, healing the heart has long been a cherished aspiration of the medical community. With the aging of the population, rheumatic valvular disease has become an increasingly common cause of cardiovascular disease prevalent, leading to approximately 250,000 deaths each year (Marijon et al., 2012). The primary causes of the disease include acute rheumatic fever, rheumatic carditis, and the inflammatory exudation and adhesion of the valve and its base. Clinicians therefore replace diseased valves with transcatheter prostheses by means of endovascular approaches such as transfemoral, transapical, transjugular, and transatrial approaches. The initial procedure of transcatheter heart valve replacement (THVR) was executed in the year 2002 (Bui et al., 2021). Since then, materials for heart valves have undergone numerous iterations, incorporating various soft segments such as polyether, polyester, polysiloxane, and polycarbonate. Due to the limitations of traditional heart valve materials. For instance, mechanical valves require lifelong anticoagulation, while biological valves degrade due to calcification within 7–8 years (Goode et al., 2025; Prabhu et al., 2025). So recent research has addressed these gaps by developing biomaterials that simulate the anisotropic strength, elasticity and regenerative potential of natural valves (Boehm et al., 2023; Snyder and Jana, 2023).

The production strategy for bio-inspired materials primarily considers two aspects: structure bionic and performance bionic. Structural biomimetics aims to simulate the physical structure of natural tissue ECM, while performance biomimetics aims to design regenerative tissue with similar properties to native valves on the basis of structure (Marino et al., 2025; Wall et al., 2010). Recent research has developed biomimetic heart valves that replicate the hierarchical structure and mechanical properties of native valves using advanced textile technologies, electrospinning, and 3D printing. These designs achieve graded stiffness, anisotropic strength, and tissue-like compliance (Boehm et al., 2023; Hussein et al., 2020; Sennewald et al., 2025). For example, textile-based valves with biomimetic fiber arrangements (e.g., PVDF, PCU) mirror native collagen and elastin alignment, achieving burst pressures >460 N/m and pressure gradients as low as 3.02 mmHg-meeting ISO-5840 standards while outperforming native tissue mechanically (Boehm et al., 2023). Similarly, Biohybrid scaffolds combine synthetic fibers with electrospun polymers to replicate the trilayered valve histology (fibrosa, spongiosa, ventricularis), achieving anisotropic elasticity (Chen et al., 2025; Freystetter et al., 2022; Yang et al., 2025). The polylactic acid/GelMA anisotropic nanofiber scaffold, fabricated by electrospinning combined with thermal stretching technology, achieved a fiber orientation degree of 0.8, which increased cell spreading efficiency from 8.6% to 62%. This scaffold successfully mimicked the mechanical microenvironment of native valves and guided orderly cell growth (Chen et al., 2026). At the level of surface modification, Functional biomimicry involves surface modification and ECM-mimetic composition. Incorporating unmodified hyaluronic acid (HA) into fibrin scaffolds reduces leaflet retraction by 25% (Lei et al., 2021). H_2_-N_2_ plasma treatment significantly enhanced the hydrophilicity and cell adhesion of PCL scaffolds without altering their mechanical properties, providing an optimized interface for the growth of human mesenchymal stem cells (Lutter et al., 2026). A more promising clinical approach involves coating elastin-like recombinamer onto thermoplastic polyurethane stent struts via dual electrospinning, with the electrospun fibers extending into the lumen to form thin leaflets. The resulting bicuspid valve exhibits extremely low platelet adhesion, no hemolysis, and excellent hemodynamic performance (Arcuti et al., 2026). At the level of surface functionalization, the biomimetic erythrocyte membrane-camouflaged nanoplatform targets endothelial cells via CD144 antibodies and continuously releases 2-deoxy-D-ribose from the PLGA core to activate the EGFR-MAPK pathway, achieving the formation of a confluent endothelial monolayer within 14 days in a rat model (Qiu et al., 2026). Compared with surface functionalization strategies, the endogenous regeneration approach goes a step further: it is no longer confined to interface modulation, but instead guides *in situ* tissue regeneration through the material itself. Acellular scaffolds like the HCCV use polycaprolactone (PCL) matrices to recruit host cells for endogenous regeneration, avoiding cell-based therapy challenges (Yacoub et al., 2023).

However, Tissue-engineered heart valves face durability challenges, as cyclic loading over years can induce damage at high-stress regions such as commissures and suture lines (Bhat et al., 2025; Mao et al., 2025; Martin and Sun, 2017). Accelerated wear testing simulates decades of use within months—polymer valves tested at high frequency have remained functional beyond 50 million cycles, although test conditions do not fully replicate physiological environments (Martin and Sun, 2017; Ponnaluri et al., 2023). Computational models, including finite element analysis, help predict stress distribution and potential crack initiation advances in materials, such as modified polymer valves, have demonstrated resistance to calcification and maintained integrity after millions of test cycles (Rotman et al., 2020). Design modifications, including stent geometry optimization and anti-calcification coatings, further contribute to extended valve durability (Kuang et al., 2020). In addition, emerging 3D-printed prototypes (e.g., TRISKELION silicone valve) achieve regurgitation fractions (22.26%) and pressure gradients (9.93 mmHg) comparable to commercial bioprostheses (Tschorn et al., 2022). Given the limitations of current testing methods, integrating accelerated wear tests, computational simulations, 3D printing technology, and real-time monitoring may provide a more reliable strategy for predicting long-term valve performance (Marino et al., 2025).

**FIGURE 6:**
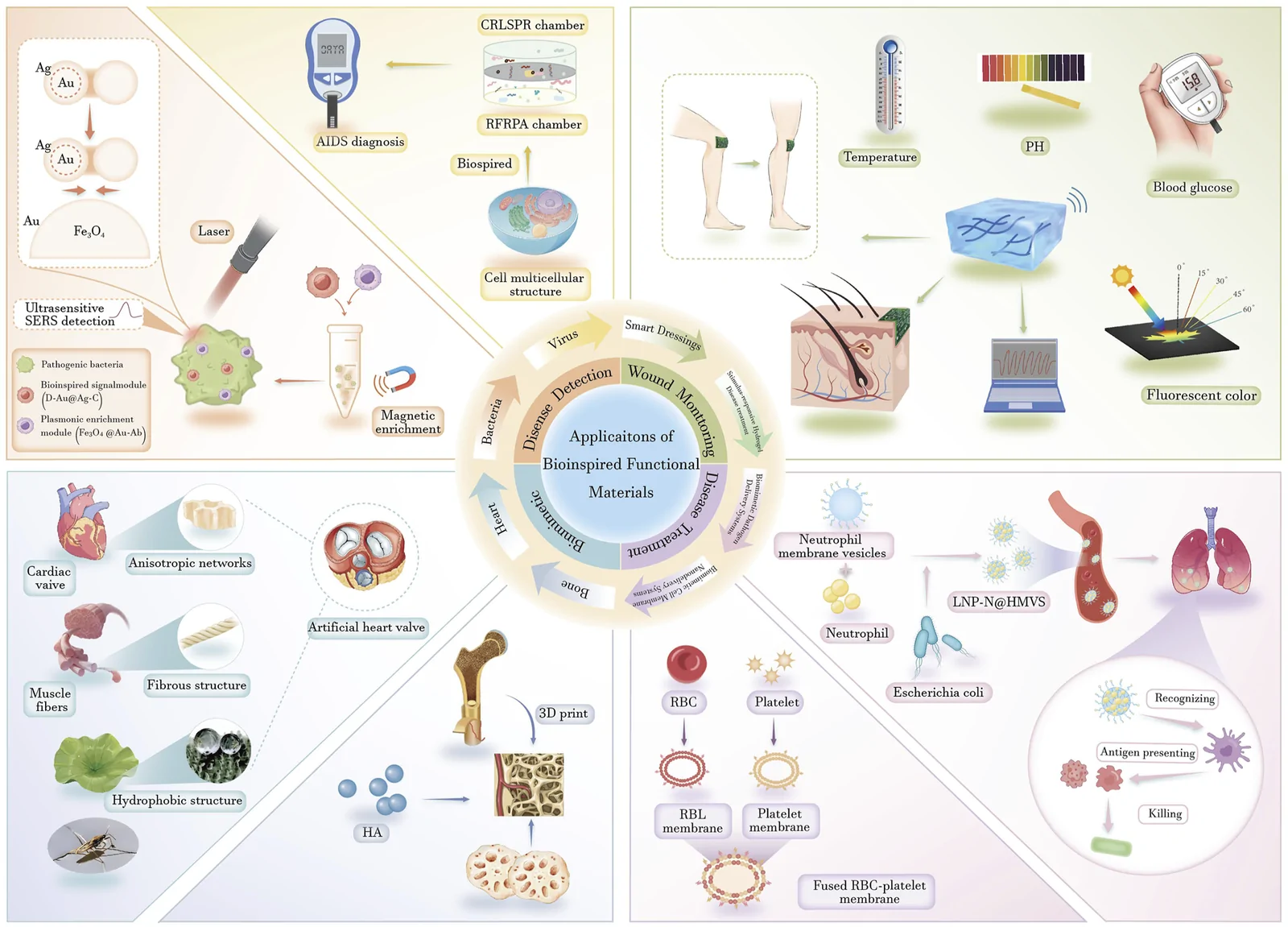
Applications of bioinspired functional materials.

Figure 6 provides an overview of the biomedical applications of bio-inspired materials discussed in this review, including disease detection, targeted therapy, and biomimetic implants, illustrating how nature-inspired design translates into functional solutions across the healthcare spectrum.

## 7 Discussion

This article reviews three primary categories of biomimetic materials: hydrogels, bioceramics, and biomimetic metals, emphasizing their design principles, structural characteristics, and biomedical applications. These examples demonstrate that biologically inspired designs can provide significant therapeutic advantages. Nonetheless, numerous challenges hinder their clinical translation. For instance, the large-scale, cost-effective production of biomimetic structures remains problematic. Additionally, the long-term metabolic pathways and immune responses associated with the degradation products of biomimetic coatings or degradable metals in the body are not yet fully understood. Furthermore, there is a lack of specialized evaluation criteria.

To address these issues, future research should concentrate on: (1) multi-scale simulation and design, which integrates computational materials science and machine learning to predict the relationship between structure and performance, thereby accelerating material selection; (2) dynamic and smart materials that can respond to changes in the body’s microenvironment, such as pH, enzymes, and mechanical signals, to facilitate on-demand treatment; (3) large-scale manufacturing, exemplified by 3D printing, which can produce complex, patient-specific designs and is also amenable to large-scale production; (4) self-decision-making bioelectronic systems that combine flexible sensors with smart materials to create a treatment system capable of autonomous perception, decision-making, and intervention; and (5) specialized evaluation standards and regulatory science aimed at establishing criteria for biomimetic materials that prioritize producibility and regulatory compliance.

## 8 Conclusion

This review systematically examines hydrogels, bioceramics, and biomimetic metals—their design principles, structural features, and applications in disease detection, targeted therapy, and implants. By mimicking nature, these materials achieve enhanced biocompatibility and functionality. Despite challenges in manufacturing and safety, integrating multidisciplinary approaches and advanced fabrication technologies will enable bio-inspired materials to transcend mere imitation, offering superior solutions for precision medicine.
